# The Liver–Heart Axis in Rheumatoid Arthritis: Associations of Liver Fibrosis, Organokines, Endothelin-1, and Cardiovascular Risk

**DOI:** 10.3390/ijms27156844

**Published:** 2026-07-30

**Authors:** Mariusz Ciołkiewicz, Anna Kuryliszyn-Moskal, Ewa Jabłońska, Wioletta Ratajczak-Wrona, Jacek Robert Janica, Włodzimierz Samborski, Piotr Adrian Klimiuk

**Affiliations:** 1 Department of Rehabilitation, Medical University of Bialystok, 15-089 Bialystok, Poland; anna.kuryliszyn-moskal@umb.edu.pl; 2 Department of Immunology, Medical University of Bialystok, 15-269 Bialystok, Poland; ewa.jablonska@umb.edu.pl (E.J.); wioletta.ratajczak-wrona@umb.edu.pl (W.R.-W.); 3 Department of Radiology, Medical University of Bialystok, 15-276 Bialystok, Poland; jrjanica@gmail.com; 4 Department of Rheumatology, Rehabilitation and Internal Diseases, Poznan University of Medical Sciences, 61-545 Poznan, Poland; wlodzimierz.samborski@ump.edu.pl; 5 Department of Rheumatology and Internal Medicine, Medical University of Bialystok, 15-276 Bialystok, Poland; piotr.klimiuk@umb.edu.pl

**Keywords:** rheumatoid arthritis, liver fibrosis, myostatin, resistin, osteoprotegerin, endothelin-1, cardiovascular risk, left ventricular diastolic dysfunction

## Abstract

Liver involvement is frequent in rheumatoid arthritis (RA) and may progress from steatosis to fibrosis, cirrhosis, or hepatocellular carcinoma. Liver fibrosis (LF) in RA is multifactorial, but the available data are limited and the impact of methotrexate (MTX) remains controversial. In this cross-sectional study, 51 RA patients (46 females; mean age of 48.8 ± 8.2 years; and a median disease duration of 12 years) were enrolled. LF was assessed using non-invasive indices (the aspartate aminotransferase-to-platelet ratio index [APRI] and fibrosis-4 index [FIB-4]) and liver stiffness measurement (LSM) using shear wave elastography. Serum endothelin-1 (ET-1) and selected organokines (namely myostatin, resistin, and osteoprotegerin) were quantified by the ELISA. Associations of the APRI, FIB-4, and LSM with organokines and endothelin-1 were analyzed using univariable and multivariable linear regression, whereas correlations with the RA-specific cardiovascular (CV) risk score (ERS-RA) and echocardiographic parameters of left ventricular diastolic dysfunction (LVDD) were assessed using Spearman’s rank correlation. Myostatin and resistin showed a significant positive and significant negative association with LSM, respectively, while FIB-4 was negatively correlated with lateral and medial e’ velocities and positively with the ERS-RA. MTX use, mean weekly dose, cumulative dose, and endothelin-1 were not associated with the APRI, FIB-4, LSM, or LVDD. These exploratory findings support the potential role of myostatin and resistin in LF-related phenotypes and suggest that FIB-4 may capture aspects of both hepatic and cardiovascular risk in RA. RA patients with elevated FIB-4 values may require echocardiographic assessment and comprehensive CV risk evaluation. In this cohort, MTX exposure and endothelin-1 were not associated with LF or LVDD.

## 1. Introduction

Liver involvement is common in patients with rheumatoid arthritis (RA) and may progress from steatosis to steatohepatitis, fibrosis, cirrhosis, or hepatocellular cancer. Liver fibrosis (LF) in RA is multifactorial, arising from both drug-related hepatotoxicity and long-standing metabolic dysfunction-associated steatotic liver disease (MASLD), which is mediated by metabolic and immune mechanisms [1,2,3,4]. Evidence regarding the impact of MTX on LF remains inconsistent. Some studies report an association between MTX use, MASLD and LF [1,2,3], whereas others find no such relationship [5,6,7].

The pathogenesis of liver fibrosis in RA remains an active area of investigation. Identifying novel biomarkers may improve early diagnosis, enable targeted interventions, and help to prevent progression.

Among potential mediators, endothelin-1, a peptide primarily synthesized by vascular endothelial cells, induces smooth muscle contraction and proliferation, as well as collagen synthesis in activated hepatic stellate cells. Inhibition of its receptor ameliorates liver fibrosis [8,9].

Growing evidence also supports the involvement of organokines in the complex mechanisms of liver fibrosis. Crosstalk among hepatokines, myokines, and adipokines appears to be important in the development of MASLD and its progression to fibrosis [10].

Myostatin, also known as growth and differentiation factor 8, is a member of the transforming growth factor-β family, predominantly secreted by skeletal muscle. It acts as a potent negative regulator of muscle growth and regeneration, contributing to sarcopenia and cachexia. Myostatin also promotes osteoclast differentiation, thereby inhibiting bone formation and driving articular bone loss in RA [11]. In RA, higher myostatin levels have been linked to disease activity, inflammatory parameters [12], and accelerated joint destruction [13]. Increasing evidence highlights the pathogenetic, prognostic, and therapeutic significance of myostatin in chronic liver diseases [14,15,16,17,18].

Resistin, an adipokine mainly expressed in bone marrow, monocytes, and macrophages, contributes to endothelial dysfunction, inflammation, vascular smooth muscle cell proliferation, and atherosclerosis [19]. In RA, serum resistin levels correlate with disease activity [20,21], duration [22], and progression [23]. In liver fibrosis, resistin has been implicated in the activation of hepatic stellate cells towards a pro-fibrogenic phenotype [24] and in the dysregulation of redox homeostasis in leukocytes [25]. Several studies support its pathogenic role in LF [26,27,28], although a few have reported hepatoprotective and anti-inflammatory effects [29].

Osteoprotegerin (OPG) is an osteokine belonging to the tumor necrosis factor receptor superfamily and is secreted by osteoblasts, lymphocytes, endothelial cells, and vascular smooth muscle cells. Through interactions with its ligands, namely RANKL (receptor activator of nuclear factor-κB ligand) and TRAIL (TNF-related apoptosis-inducing ligand), OPG inhibits osteoclastogenesis, bone resorption, and apoptosis. Dysregulation of the OPG/RANKL/TRAIL axis is implicated in RA pathogenesis, promoting inflammation, osteoclast activation and bone erosion [30]. Several studies have reported elevated OPG levels in RA and associations with disease duration, activity and severity [31,32]. Recent findings suggest that the OPG/RANKL/RANK axis also contributes to hepatic steatosis, inflammation, and fibrosis [33,34,35].

Building on our previous work, in which we demonstrated that MASLD and elevated liver fat scores (the Fatty Liver Index and Hepatic Steatosis Index) are common in RA and relate to organokines, cardiovascular risk scores, and LVDD [36], we now extend this concept to hepatic fibrosis. While liver steatosis reflects early stages of cardiohepatic involvement, fibrosis represents a more advanced and prognostically relevant phenotype along the liver–heart axis. Therefore, the aim of this cross-sectional study was to investigate the associations of non-invasive liver fibrosis indices and liver stiffness with selected organokines, endothelin-1, RA-specific cardiovascular risk, and the echocardiographic parameters of left ventricular diastolic dysfunction in patients with rheumatoid arthritis. In addition, we sought to assess the relationship between methotrexate exposure and liver fibrosis in this cohort.

## 2. Results

### 2.1. Characteristics of the Study Population

The study included 51 patients with rheumatoid arthritis (46 females and 5 males; mean age 48.8 ± 8.2 years). The median disease duration was 12.0 years (IQR 4.95–20.25), with moderate clinical activity (mean CDAI 25.0 ± 12.1) and low inflammatory activity (median hsCRP 2.88 mg/L; IQR 1.06–6.19).

In total, 49 patients (96.1%) received conventional synthetic disease-modifying antirheumatic drugs (csDMARDs), including methotrexate in 46 patients (90.2%) at a mean weekly dose of 16.2 ± 4.4 mg and a median cumulative dose of 6100 mg (IQR 2400–10,935). Biological DMARDs were used in 32 patients (62.7%), corticosteroids in 11 patients (21.6%; mean prednisone-equivalent dose 6.2 mg/day), and NSAIDs in 28 patients (54.9%). The median daily alcohol consumption was 0.67 g (IQR 0.00–1.33), and only one female patient reached the maximum dose of 20 g/day. Twelve (23.5%) patients had hypertension, and one patient (1.9%) had diabetes.

### 2.2. Laboratory Findings

Eleven patients (21.6%) had elevated liver enzymes. The median AST, ALT, and GGT levels were within normal reference ranges, at 20 U/L (IQR 15.0–25.0), 19.0 U/L (IQR 15.0–32.0), and 10.9 U/L (IQR 8.2–16.8), respectively. The median NLR value was low at 1.78 (IQR 1.13–2.26). The median interleukin-6 level was 5.06 pg/mL (IQR 1.23–9.79), and the median endothelin-1 level was 0.84 pg/mL (IQR 0.74–1.19). Regarding organokines, the median levels of myostatin, resistin, and osteoprotegerin were 2435.4 pg/mL (IQR 2001.8–3444.9), 8.05 ng/mL (IQR 5.73–10.42), and 256.0 pg/mL (IQR 216.6–299.4), respectively.

### 2.3. Liver Fibrosis Scores

The aspartate aminotransferase-to-platelet ratio index (APRI) was ≥0.5 in four patients (7.8%), with a median value of 0.20 (IQR 0.10–0.30). The fibrosis-4 (FIB-4) score indicated liver fibrosis (≥1.3) in five patients (9.8%), with a median value of 0.87 (IQR 0.54–1.07).

Elastography demonstrated increased liver stiffness consistent with liver fibrosis (≥6.5 kPa) in only one patient (1.9%), with a median value of 3.94 kPa (IQR 3.65–4.40).

### 2.4. Echocardiographic Findings

The mean peak E and A velocities, lateral and septal mitral annular velocities, E/A ratio, median E/e′ ratio, and mean left atrial size parameters (LAD, LAV, and LAVI) were all within normal reference ranges. Ten patients (19.6%) met the criteria for LVDD, and in all of them, it was mild (grade I LVDD). The laboratory findings, liver fibrosis indices, and echocardiographic parameters of LVDD, and ERS-RA score are summarized in Table 1.

### 2.5. Pairwise Agreement Between Liver Fibrosis Scores

**Figure 1:**
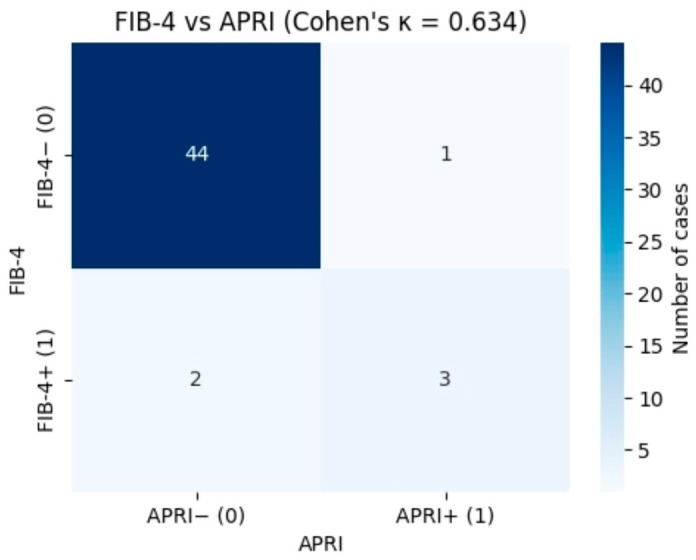
Pairwise agreement between liver fibrosis scores. Cohen’s κ = 0.63 indicates substantial agreement between the APRI and FIB-4 scores. Abbreviations: APRI, aspartate aminotransferase-to-platelet ratio index; FIB-4, fibrosis-4.

Agreement between the APRI and FIB-4 in predicting liver fibrosis was substantial, with 94% raw agreement and κ = 0.63. Figure 1 visualizes the pairwise agreement between these liver fibrosis scores.

### 2.6. Associations of Liver Fibrosis Scores with Clinical Parameters

#### 2.6.1. Age, Sex, Disease Activity and Duration

Age and female sex were not associated with the APRI or LSM (APRI: *p* = 0.57 and *p* = 0.87; LSM: *p* = 0.37 and *p* = 0.34, respectively). Female sex was also not associated with FIB-4 (*p* = 0.81). The lack of statistically significant associations between female sex and the APRI, FIB-4, or LSM should be interpreted with caution because of the marked predominance of women in the cohort and the small number of male participants.

Each one-year increase in age was associated with a 0.03-point increase in FIB-4 (*p* < 0.001). No relationships were observed between clinical disease activity assessed by the CDAI and APRI (*p* = 0.68), FIB-4 (*p* = 0.85), or LSM (*p* = 0.87). Disease duration was not associated with the APRI (*p* = 0.95) or FIB-4 (*p* = 0.34), with only a weak negative, non-significant association suggested for LSM (β = −0.03, *p* = 0.07).

#### 2.6.2. Alcohol Use and RA Treatment

Neither MTX use (*p* = 0.62, *p* = 0.72, and *p* = 0.14, respectively), median weekly MTX dose (*p* = 0.42, *p* = 0.15, and *p* = 0.95, respectively), nor cumulative MTX dose (*p* = 0.39, *p* = 0.30, and *p* = 0.70, respectively) were associated with the APRI, FIB-4, or LSM.

Biologic therapy was not associated with liver fibrosis according to any score (APRI: *p* = 0.47; FIB-4: *p* = 0.24; LSM: *p* = 0.28). Glucocorticoid use was associated with a 0.31-point decrease in FIB-4 (*p* = 0.02), whereas no associations were observed with the APRI (*p* = 0.42) or LSM (*p* = 0.45). NSAID use was not associated with liver fibrosis, as reflected by the APRI, FIB-4, or LSM (*p* = 0.47, *p* = 0.66, and *p* = 0.76, respectively). There were no relationships between daily alcohol consumption and liver fibrosis scores (APRI: *p* = 0.38; FIB-4: *p* = 0.65) or liver stiffness measurement (*p* = 0.15).

#### 2.6.3. Inflammatory Markers

Regarding inflammatory markers, each 1 mg/dL increase in hsCRP was associated with a 0.004-point increase in the APRI (*p* < 0.001), whereas no associations were observed with FIB-4 (*p* = 0.16) or LSM (*p* = 0.31). There was a trend toward a positive association between IL-6 and the APRI (beta = 0.001, *p* = 0.09), but this was not the case with FIB4 (*p* = 0.71) or LSM (*p* = 0.37). NLR was not related to liver fibrosis (APRI: *p* = 0.57; FIB-4: *p* = 0.65; LSM: *p* = 0.99).

### 2.7. Associations of Liver Fibrosis Indices with Endothelin-1 and Organokines

Endothelin-1 was not associated with any of the liver fibrosis indices (APRI: *p* = 0.28; FIB-4: *p* = 0.39; LSM: *p* = 0.76). None of the investigated organokines were associated with the APRI (myostatin: *p* = 0.53; resistin: *p* = 0.69; OPG: *p* = 0.19). A trend toward a positive association with FIB-4 was observed for OPG (β = 0.001, *p* = 0.09), with no association observed for myostatin (*p* = 0.24) or resistin (*p* = 0.40).

Myostatin was significantly positively associated (β = 0.26, *p* = 0.04) and resistin negatively associated (β = −0.14, *p* = 0.002) with LSM, while no association was observed for OPG (*p* = 0.57). After adjustment for age, sex, and MTX use in multivariable regression models, resistin and myostatin were the only variables independently associated with LSM.

Because the numbers of patients with liver fibrosis defined by the APRI, FIB-4, and LSM were small, the receiver operating characteristic (ROC) analyses evaluating the predictive value of inflammatory markers, endothelin-1, and organokines had limited statistical power.

**Figure 2:**
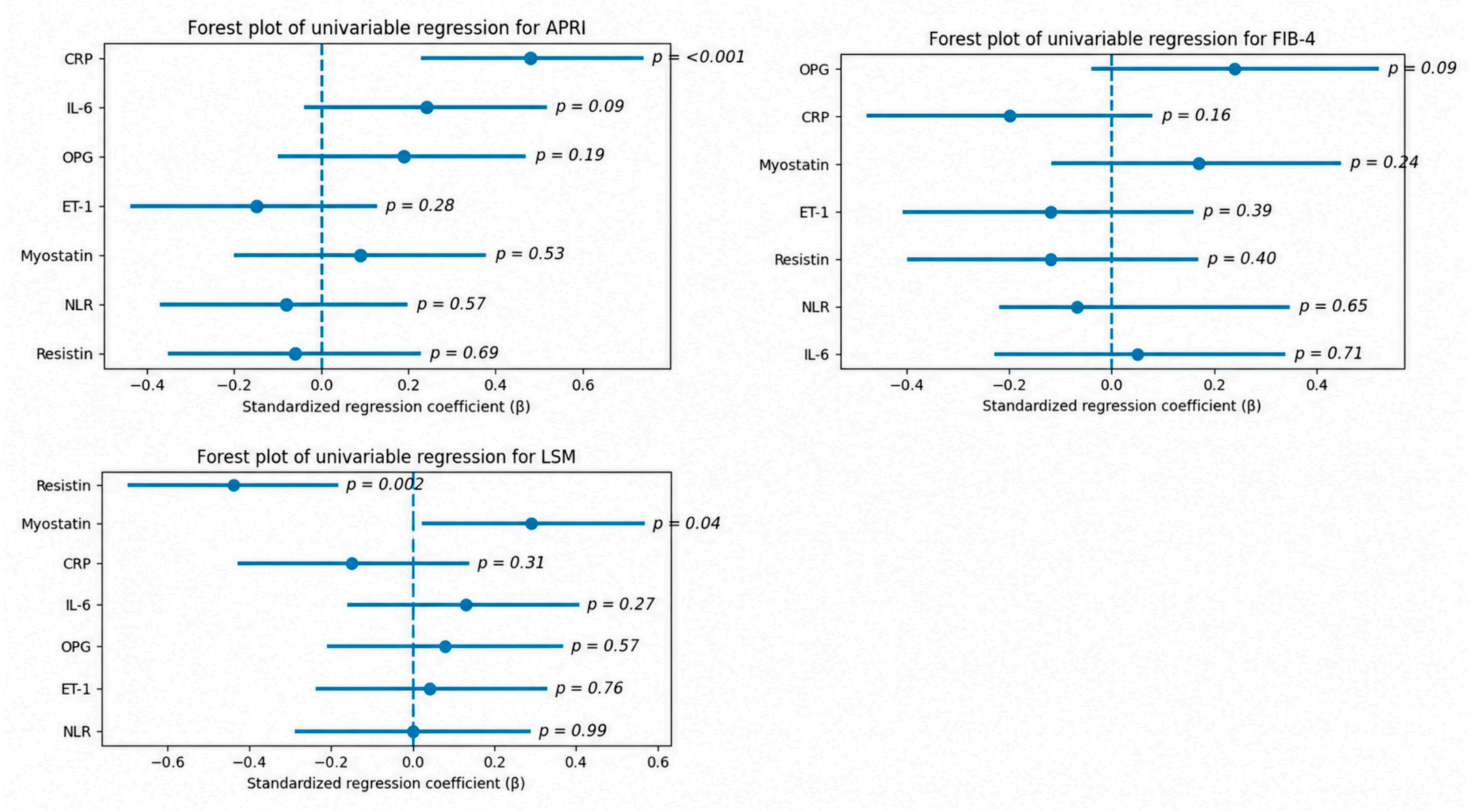
Forest plots of standardized β coefficients with 95% confidence intervals from univariable linear regression models for the APRI, FIB-4, and LSM. Positive β values indicate direct associations, whereas negative β values indicate inverse associations. Associations with *p* < 0.05 were considered statistically significant, The dashed vertical line marks β = 0 (no effect);only estimates with 95% CIs that did not cross the zero line were deemed significant. CRP showed a statistically significant positive association with the APRI (*p* < 0.001), whereas resistin (positive association) and myostatin (inverse association) were significantly associated with LSM (*p* = 0.002 and *p* = 0.04, respectively); no significant associations were observed for the other parameters. Abbreviations: APRI, aspartate aminotransferase-to-platelet ratio index; CRP, C-reactive protein; ET-1, endothelin-1; FIB-4, fibrosis-4; IL6, interleukin-6; LSM, liver stiffness measurement; OPG, osteoprotegerin; NLR, neutrophil-to-lymphocyte ratio.

The results of univariable linear regression analyses for FIB-4, the APRI and LSM are presented in Tables S1 and S2 in the Supplementary Materials. Forest plots of standardized β coefficients from univariable linear regression models for FIB-4, the APRI and LSM are presented in Figure 2.

### 2.8. Left Ventricular Diastolic Dysfunction, RA Treatment, and Liver Fibrosis Indices

Neither MTX use (OR 0.97, *p* = 0.98), median weekly MTX dose (OR 1.03, *p* = 0.72), nor cumulative MTX dose (OR 1.00, *p* = 0.11) was associated with the odds of LVDD. Use of biologic DMARDs (OR 0.86, *p* = 0.84), NSAIDs (OR 3.8, *p* = 0.12), and corticosteroids (OR 0.89, *p* = 0.89) was not associated with the occurrence of LVDD.

None of the liver fibrosis indices were associated with increased odds of LVDD (APRI: OR 1.99, *p* = 0.78; FIB-4: OR 2.41, *p* = 0.30; LSM: OR 1.91, *p* = 0.13).

### 2.9. LVDD, Endothelin-1 and Organokines

Resistin showed a trend toward a negative association with LVDD (OR 0.77, *p* = 0.07), whereas osteoprotegerin showed a trend toward a positive association (OR 1.007, *p* = 0.08). There was no relationship between myostatin (OR 1.00, *p* = 0.26) or endothelin-1 (OR 2.51, *p* = 0.25) and LVDD risk. Table S3 in the Supplementary Materials summarizes the results of the logistic regression analysis for LVDD.

### 2.10. Liver Fibrosis Scores and Echocardiographic LVDD Parameters

FIB-4 was negatively correlated with e’ lat and e’ med., indicating that a higher risk of liver fibrosis was associated with worse left ventricular diastolic function. The correlation was moderate for e’ lat (r = −0.42, *p* = 0.002) and weak-to-moderate for e’ med. (r = −0.32, *p* = 0.023). A trend toward a negative correlation was observed with E/A (*p* = 0.06), and a trend toward a positive correlation was observed with E/e’ (*p* = 0.09). Neither the APRI nor LSM was correlated with any LVDD parameters.

### 2.11. Correlations Between Liver Fibrosis Indices and Cardiovascular Risk

Among liver fibrosis scores, only FIB-4 showed a moderate positive correlation with the ERS-RA (r = 0.42, *p* = 0.002).

## 3. Discussion

Our findings complement our previous report on MASLD and liver fat scores in RA, in which selected organokines (fatty acid-binding protein 4 and fibroblast growth factor 21) showed diagnostic relevance for hepatic steatosis and the Fatty Liver Index demonstrated cardiovascular relevance [36]. In the present study, we extend these observations from steatosis to fibrosis-related phenotypes, highlighting myostatin and resistin as potential organokine mediators and suggesting that FIB-4 may capture aspects of both hepatic and cardiovascular risk in RA.

To our knowledge, the present results are the first to demonstrate a positive association between FIB-4 and cardiovascular risk assessed using an RA-specific score (ERS-RA).

In a cross-sectional study by Ferraz-Amaro et al. [37] involving 465 RA patients (mean age: 55 years; 81% women; median disease duration: 8 years), FIB-4 was significantly associated with higher CV risk, estimated by SCORE2 (Systematic Coronary Risk Evaluation 2), but not with carotid atherosclerosis. In that cohort, 20% of patients had FIB-4 values within the indeterminate or high-risk range for liver fibrosis, and FIB-4 was not correlated with RA characteristics, including disease activity. In our younger population, with a higher proportion of women and a 50% longer disease duration, FIB-4 values indicated liver fibrosis in 9.8% of patients and were significantly associated with CV risk, as assessed by the ERS-RA, but again showed no relationship with RA characteristics.

Our results demonstrating an association between FIB-4 and increased CV risk are in line with the study by Saidi et al. [38], which included 326 RA patients without cardiovascular disease or type 2 diabetes (68.4% female; mean age: 53 years; mean BMI: 26.5 kg/m^2^), in whom elevated FIB-4 scores were associated with higher blood pressure, increased carotid intima–media thickness, and elevated atherogenic remnants.

Evidence regarding the relationship between FIB-4 and the echocardiographic parameters of LVDD is currently lacking. In the present study, we observed negative correlations between FIB-4 and both medial and lateral e’ velocities, as well as trends towards a positive correlation with the E/e’ ratio and a negative correlation with the E/A ratio. Taken together with the association between FIB-4 and increased CV risk, as assessed by the ERS-RA, these findings suggest that FIB-4 may be a useful tool in RA for identifying patients at increased cardiovascular risk and for supporting more intensive risk factor control and earlier initiation of cardioprotective treatment.

Our study revealed a significant inverse relationship between resistin and liver stiffness and a trend toward a negative association with LVDD, suggesting a potential beneficial effect of resistin with respect to hepatic and myocardial fibrosis. This observation is consistent with findings by Xu et al. [29] concerning a murine acute-on-chronic liver failure model, in which resistin exerted marked hepatoprotective and anti-inflammatory effects. Neutralization of resistin enhanced hepatocyte apoptosis, exacerbated liver injury, and increased systemic cytokine levels, whereas administration of recombinant resistin alleviated hepatic injury, reduced inflammatory cytokine levels, and improved survival.

On the other hand, several studies have highlighted a negative role of resistin in liver fibrosis. In patients with chronic hepatitis B, Custovic et al. [26] reported a positive correlation between serum resistin levels and the degree of liver fibrosis, as assessed by FIB-4 and the APRI. Similarly, Meng et al. [27] showed that high serum resistin levels in HBV-infected individuals were associated with intrahepatic inflammation and necrosis and were positively correlated with serum TGF-β1 levels in cirrhotic patients, suggesting that resistin may serve as an index of liver disease severity. Da Silva et al. [39] observed higher resistin concentrations in patients with liver cirrhosis compared with controls, although resistin levels were not related to survival. Further studies are needed to clarify the role of resistin in patients with liver fibrosis.

Evidence regarding the role of myostatin in liver fibrosis among patients with rheumatoid arthritis is lacking. In our study, we found a significant positive association between myostatin and liver stiffness. It has been proposed that NAFLD-related fibrosis and sarcopenia share common pathophysiological pathways and that myostatin signaling may promote muscle wasting in patients with NAFLD-related fibrosis [40]. In a study of 301 patients afflicted by the spectrum of alcohol-associated liver disease [15], higher myostatin levels were linked to fibrosis, systemic inflammation, organ failure, disease progression, and mortality. In addition, Kim et al. showed that myostatin predicted the development of hepatocellular carcinoma in patients with alcoholic cirrhosis [17]. Conversely, Ruiz-Margáin et al., in a study of patients with liver cirrhosis, observed a stepwise decrease in myostatin levels with disease progression [16], while in advanced chronic liver failure, Skladany et al. [18] found myostatin to be negatively correlated with CRP, with lower myostatin levels predicting worse survival and higher levels reflecting sarcopenia in men. In our cohort, myostatin was significantly associated with liver stiffness values but not with inflammatory markers (hsCRP, IL-6, and NLR), and only one patient had liver stiffness values within the range consistent with liver fibrosis. We also did not assess body composition or muscle strength in relation to liver stiffness, liver fibrosis scores, or myostatin, which precluded evaluation of the impact of sarcopenia. This remains an important area for further investigation.

Despite the fact that osteoprotegerin is linked to fibrosis and has even been incorporated into serum panels for the diagnosis of liver fibrosis [34,35], we found no associations with the APRI score or liver stiffness measurements and a trend only toward a positive association with FIB-4. Studies including a larger number of patients are warranted to clarify these relationships.

In our study, we observed no relationship between ET-1 and liver fibrosis scores, liver stiffness, methotrexate use, and left ventricular diastolic dysfunction. This may reflect the small number of patients with APRI, FIB-4, or LSM values indicative of liver fibrosis. Emerging data nevertheless support the involvement of ET-1 in murine and human liver fibrosis and cirrhosis [8], and its contribution to liver fibrosis in rheumatoid arthritis therefore merits further investigation.

In the present study, we observed no relationship between MTX use, mean weekly MTX dose, cumulative MTX dose, and liver fibrosis. This is consistent with the findings of Kim et al. [5], who reported substantial liver fibrosis, defined as liver stiffness > 8.6 kPa by SWE, in approximately 5% of 187 MTX-treated RA patients, with fibrosis associated only with higher BMI values and not with cumulative MTX dose, suggesting that other factors may play a more important role in LF. In our study, which assessed only mild liver fibrosis, observed in 1.9% of patients, we found no association between BMI and LSM values (*p* = 0.17) but a positive association with myostatin and a negative association with resistin.

Similarly, in a cross-sectional study of 119 RA patients assessed with acoustic radiation force impulse imaging elastography, Feuchtenberger et al. [6] observed no increased rate of LF among MTX-treated RA patients compared with MTX-naïve individuals. The mean cumulative methotrexate dose in the MTX-exposed group was 3602 mg, whereas in our study, the mean cumulative dose was higher by 72%.

In the study by Darabian et al. [41], conducted with a cohort of 520 patients with arthritis (mean age: 58.6 years; 63% female; mean BMI: 27.6 kg/m^2^; mean cumulative MTX dose: 3142.6 mg), the prevalence of F3-F4 liver fibrosis was 2.7%. Consistent with our findings, no significant correlation between cumulative MTX dose and liver stiffness was observed, even at high MTX doses. In contrast to our results, BMI, male sex, and age were statistically significant determinants of liver stiffness in that cohort.

In another study of 170 patients with RA [7], 83% were women, the mean age was 59 years, and the mean disease duration was 15 years. The FIB-4 index, reflecting liver fibrosis, was low (mean: 1.24) in 71% of patients, with values below 1.54, and did not differ significantly between patients currently receiving MTX, those previously treated with MTX, and MTX-naïve individuals. The authors also found no correlation between FIB-4 values and either cumulative MTX dose or MTX treatment duration. Higher FIB-4 values were associated with male sex, low disease activity, and treatment with leflunomide or tocilizumab. In our cohort, patients were younger, they had shorter disease duration, and a higher proportion of patients were women; the median FIB-4 value was lower (0.87) and was not associated with any treatment, with the exception of glucocorticoids, probably due to their effect on increasing platelet count. We found no relationships with sex or clinical disease activity.

On the other hand, in a cross-sectional study of 75 RA patients (92% female; mean age: 47.2 years; mean BMI: 24.8 kg/m^2,^; mean waist circumference: 91.6 cm) by Bafna et al. [1], the median MTX treatment duration and cumulative dose were 336 weeks and 6300 mg, respectively, and the mean liver stiffness was 5.22 kPa. Sixteen percent of patients had a Fibroscan score ≥ 7.1 kPa, indicating liver fibrosis, and Fibroscan values correlated significantly with cumulative MTX dose. In our study, the age, proportion of women, and cumulative MTX dose were similar, with a slightly higher BMI; however, the median liver stiffness, measured by SWE, was approximately 25% lower, and the occurrence of liver fibrosis was about eight times less frequent.

An additional consideration is the potential influence of menopausal status and estrogen-related hormonal changes. Given the predominantly female composition of our cohort and the mean age of 48.8 years, participants may have included both premenopausal and peri- or postmenopausal women. The menopausal transition is associated with changes in estrogen exposure that may affect immune regulation, inflammatory processes, body composition, metabolic homeostasis, and cardiovascular risk [42], all of which may be relevant to RA, diastolic dysfunction, and liver fibrosis-related phenotypes. However, the effects of estrogen on autoimmune and rheumatic diseases are complex and remain incompletely understood, with potentially divergent effects depending on hormonal status and disease context. As menopausal status, hormone replacement therapy, and circulating estrogen concentrations were not assessed in our study, their potential contribution to the observed associations could not be evaluated. Future studies should incorporate detailed reproductive and hormonal data to clarify whether menopausal status or estrogen-related changes modify the relationships between RA, liver fibrosis-related measures, and cardiovascular risk.

A further point to consider is the potential interaction between MTX exposure and steroid hormone status. Recent evidence indicates that MTX treatment may be associated with partial restoration of steroid homeostasis in postmenopausal women with RA, whereas glucocorticoid use may exacerbate endocrine disruption [43]. Because steroid hormones can influence immune responses, inflammatory activity, metabolic homeostasis, and cardiovascular risk, MTX-related hormonal effects could potentially contribute to the heterogeneity of hepatic and cardiovascular phenotypes. In the present study, however, circulating steroid hormone concentrations were not assessed; therefore, the potential relationship between MTX exposure and hormonal status could not be evaluated. Consequently, the lack of significant associations between MTX exposure and liver fibrosis-related measures should be interpreted cautiously. Further prospective studies incorporating detailed hormonal profiling, menopausal status, and MTX exposure are warranted to clarify the potential interplay between MTX, steroid hormones, and hepatic and cardiovascular outcomes in RA.

The present study has several limitations. The relatively small sample size (*n* = 51) and the cross-sectional, single-center design limit the statistical power and generalizability of the findings. Although the predominance of women is consistent with the known epidemiology of RA, the marked female predominance in our cohort (46/51, 90.2%) was greater than that generally reported in RA populations, resulting in a very small number of male participants. Consequently, the study had limited power to assess sex-related associations, and the absence of statistically significant associations between sex and the APRI, FIB-4 or LSM should not be interpreted as evidence of no sex effect. This sex imbalance also limits the generalizability of our findings, particularly to male patients with RA, and larger studies with a more balanced sex distribution are required to evaluate potential sex-specific differences in liver fibrosis-related outcomes. Moreover, menopausal status, hormone replacement therapy, and steroid hormone profiles, including circulating estrogen concentrations, were not assessed; therefore, the potential influence of estrogen-related hormonal changes on the observed liver fibrosis-related and cardiovascular associations, as well as potential MTX-related effects on hormonal status and these outcomes, could not be evaluated.

Another important limitation is that only a few patients in our RA cohort had liver fibrosis scores indicative of fibrosis, and only one had LSM values within the fibrosis range. This precluded a meaningful assessment of the diagnostic performance of the studied markers for liver fibrosis using receiver operating characteristic analyses and logistic regression. Moreover, all detected cases of liver fibrosis were of mild severity. Finally, the lack of body composition analysis and muscle strength assessment prevented us from evaluating the impact of sarcopenia on liver stiffness and liver fibrosis scores.

The diagnostic and prognostic value of resistin and myostatin for predicting the development of liver fibrosis in patients with rheumatoid arthritis warrants further investigation. Incorporating FIB-4 assessments into routine clinical practice may help identify patients with increased cardiovascular risk and abnormal LVDD parameters, thereby facilitating earlier implementation of cardiovascular risk management strategies and timely treatment.

Taken together, the MASLD-focused analysis and the current fibrosis-focused study support the concept of a liver–heart axis in RA, whereby both liver fat and fibrosis indices relate to subclinical cardiac dysfunction and cardiovascular risk scores. Moreover, different stages of liver involvement in RA may be characterized by different biological profiles.

## 4. Materials and Methods

### 4.1. Study Population and Design

A total of 51 consecutive patients with rheumatoid arthritis (RA) aged > 18 years, who met the 2010 American College of Rheumatology classification criteria [44] and were on a stable treatment regimen for at least 3 months, were enrolled at the Department of Rehabilitation, Medical University of Bialystok, Poland. The study protocol was approved by the Bioethics Committee of the Medical University of Bialystok (approval no. R-I-002/234/2016; 30 June 2016 and R-I-002/328/2017; date of approval 28 September 2017), and all procedures adhered to the Declaration of Helsinki. Written informed consent was obtained from all participants prior to study inclusion.

### 4.2. Exclusion Criteria

Patients who were pregnant or patients with daily alcohol consumption exceeding 20 g (women) or 30 g (men), overlap syndrome, clinical evidence of viral/autoimmune hepatitis, drug-induced liver disease, use of high-dose statins, and other medications with a significant impact on liver function, active cancer, heart failure, or advanced kidney disease were excluded.

### 4.3. Clinical Assessments

Demographic and anthropometric data (sex, age, height, and weight) were recorded, with body mass index (BMI) calculated as weight (kg)/[height (m)]^2^. Medical history included RA duration and treatment (the weekly and cumulative methotrexate doses were recorded), presence and treatment of comorbidities, smoking status, and daily ethanol intake. Disease activity was evaluated using the clinical disease activity index (CDAI). Participant characteristics are summarized in Table 2.

### 4.4. Laboratory Measurements

Analyses comprised complete blood count, ALT (alanine aminotransferase), AST (aspartate aminotransferase), GGT (gamma-glutamyl transferase), estimated glomerular filtration rate (eGFR via MDRD equation), rheumatoid factor, and high-sensitivity C-reactive protein (hsCRP) (Roche Diagnostics, Rotkreuz, Switzerland). The neutrophil-to-lymphocyte ratio (NLR) was calculated by dividing the absolute neutrophil count by the absolute lymphocyte count.

Serum concentrations of interleukin-6 (IL-6), myostatin, resistin, endothelin-1 (R&D Systems, Minneapolis, MN, USA), and osteoprotegerin (Abcam, Waltham, MA, USA) were measured in samples stored at −80 °C using commercially available ELISA kits, in accordance with the manufacturers’ instructions.

### 4.5. Liver Fibrosis Assessment

In accordance with current European Association for the Study of the Liver guidelines (EASL) [45], simple, non-invasive clinical indices, such as the aspartate aminotransferase-to-platelet ratio index (APRI) and fibrosis-4 (FIB-4), were applied as first-line tools to assess the risk of liver fibrosis. Further evaluation included liver stiffness measurement using elastography as an imaging modality.

According to the METAVIR staging system recommended by the EASL, stage F0 corresponds to a normal liver without fibrosis, stages F1 to F3 reflect mild, moderate, and advanced fibrosis, respectively, and stage F4 indicates cirrhosis.

The APRI score, originally validated for the assessment of fibrosis progression in hepatitis C virus infection [46], was calculated using the following formula: [(AST/upper limit of the normal AST range) × 100]/platelet count. The dedicated online calculator [47] (accessed on 8 December 2025) was used. An APRI value < 0.5 indicates a low risk of liver fibrosis, 0.5–1.5 reflects a moderate risk, and >1.5 suggests a high risk corresponding to advanced fibrosis stages (F3–F4) [45].

The FIB-4 index, another validated clinical score for the estimation of liver fibrosis [48], particularly in patients with HIV/HCV coinfection, was calculated according to the formula: [(age × AST)/(platelet count × √ALT)]. The calculation was performed using the online tool [49] (accessed on 8 December 2025). FIB-4 values < 1.3 denote a low risk of advanced liver fibrosis, values between 1.3 and 2.67 indicate a moderate risk, and values > 2.67 correspond to a high risk of advanced fibrosis (F3–F4).

### 4.6. Cardiovascular Risk Assessment

The ERS-RA (Expanded Cardiovascular Risk Prediction Score for Rheumatoid Arthritis), which estimates 10-year cardiovascular morbidity and incorporates conventional risk factors (sex, hyperlipidaemia, hypertension, diabetes, and current smoking) as well as RA-specific variables (disease duration > 10 years, disease activity assessed by CDAI, disability assessed by mHAQ, and current corticosteroid use), was calculated using a publicly available Excel macro [50].

### 4.7. Liver Stiffness Measurement (LSM)

Liver stiffness was determined using shear wave elastography (SWE) with the point quantification technique (Elast-PQ, EPQ) [51]. All measurements were performed by an experienced ultrasonography radiologist using an EPIQ 7 ultrasound system (Philips, Bothell, WA, USA, software version 6.3.2.2), equipped with a C5–1 convex probe. Examinations were conducted after at least four hours of fasting, with patients positioned supine and the right arm maximally abducted to provide intercostal access to the right hepatic lobe, in accordance with current recommendations [51]. Ten independent elastography measurements (in kPa) were obtained at least 1 cm below the liver capsule, avoiding large vessels, and the median of these values was recorded. Measurement reliability was confirmed by an interquartile range-to-median ratio (IQR/M) < 30%. In line with the thresholds proposed by Bauer et al. [52], liver stiffness values < 6.0 kPa reliably excluded fibrosis, whereas values ≥ 6.5 kPa and ≥6.9 kPa indicated significant (≥F2) and advanced (≥F3) fibrosis, respectively.

Transthoracic echocardiography was performed by an experienced cardiologist using a Philips ClearVue 550 system (Philips Healthcare, Best, The Netherlands) in accordance with the recommendations of the American Society of Echocardiography and the European Association of Cardiovascular Imaging [53]. Left ventricular diastolic dysfunction (LVDD) parameters assessment included: left atrial diameter (LAD, parasternal long-axis), left atrial volume index (LAVI; apical 4-/2-chamber, modified Simpson’s method, indexed to BSA), mitral inflow E/A ratio (pulsed-wave Doppler), septal/lateral e’ velocities (tissue Doppler), E/e’ ratio (mean e’), and tricuspid regurgitation velocity (apical 4-chamber). LVDD was classified per current guidelines [54].

### 4.8. Statistical Analysis

Analyses used R (the R Foundation for Statistical Computing, Vienna, Austria, version 4.4.2) and STATISTICA (StatSoft Polska, Kraków, Poland, version 13.3) with α = 0.05. Continuous variables were assessed for normality using the Shapiro–Wilk test, skewness and kurtosis, and for homogeneity of variances using Levene’s test; normally distributed variables were expressed as mean ± SD, and non-normally distributed variables as median (IQR). Linear univariable regression and multivariable regression with a backward stepwise selection method were applied to quantitative dependent variables, whereas logistic regression was used for categorical outcomes. Spearman’s rank correlation was applied for nonparametric variables. Pairwise agreement between categorized liver fibrosis scores was assessed using Cohen’s kappa statistic.

## 5. Conclusions

In conclusion, our data show that myostatin was significantly positively associated with liver stiffness measurements, whereas resistin showed a negative association. Patients with increased myostatin levels may therefore benefit from further evaluation for liver fibrosis, including elastography. FIB-4 was significantly associated with abnormal echocardiographic parameters of LVDD and with increased RA-specific CV risk, as assessed by the ERS-RA, suggesting that elevated FIB-4 values warrant echocardiographic assessment and comprehensive CV risk evaluation in RA patients. Neither methotrexate use, mean weekly dose, cumulative dose, nor ET-1 was associated with liver fibrosis or LVDD.

However, given the cross-sectional design of the present study, these findings should be considered hypothesis-generating rather than as evidence of the biological significance of myostatin and resistin. The diagnostic and prognostic value of these biomarkers in predicting liver fibrosis in patients with rheumatoid arthritis warrants further investigation.

## Figures and Tables

**Table 1:** Laboratory findings, selected organokines, endothelin-1, liver fibrosis indices, echocardiographic parameters of LVDD, and ERS-RA score.

Variable	Mean ± SD/Median (IQR)
Total cholesterol, mg/dL	188.00 (159.50–206.50)
LDL, mg/dL	93.41 ± 31.56
HDL, mg/dL	55.58 ± 16.32
TG, mg/dL	90.00 (73.00–139.50)
AST, U/L	20 (15.00–25.00)
ALT, U/L	19.00 (15.00–32.00)
GGT, U/L	10.90 (8.20, 16.80)
hsCRP, mg/L	2.88 (1.06–6.19)
Il-6, pg/mL	5.06 (1.23; 9.79)
NLR	1.78 (1.13; 2.26)
eGFR, mL/min/1.73 m^2^	92.08 ± 14.85
Endothelin-1, pg/mL	0.84 (0.74; 1.19)
Myostatin, ng/mL	2.44 (1.96; 3.45)
Resisitin, ng/mL	8.05 (5.73; 10.42)
Osteoprotegerin, pg/mL	256.04 (216.59; 299.40)
APRI	0.20 (0.10; 0.30)
FIB-4	0.87 (0.54; 1.07)
LSM, kPa	3.94 (3.65; 4.40)
E, cm/s	65.5 ± 13.2
A, cm/s	57.49 ± 13.87
E/A	1.20 ± 0.35
e’ lat, cm/s	13.25 ± 3.14
e’ med., cm/s	11.29 ± 2.45
E/e’	5.20 (4.60–6.05)
LAD, mm	35.16 ± 4.77
LAV mL	43.1 ± 14.6
LAVI, mL/m^2^	23.82 ± 6.83
ERS-RA, %	4.10 (2.61–6.70)

Data are presented as mean ± SD for normally distributed variables or as median (IQR) for non-normally distributed variables. Abbreviations: A, peak A-wave velocity; ALT, alanine aminotransferase; APRI, aspartate aminotransferase-to-platelet ratio index; AST, aspartate aminotransferase; E, peak E-wave velocity; e’ lat, lateral mitral annular velocity; e’med, septal mitral annular velocity; eGFR, estimated glomerular filtration rate; ERS-RA, expanded cardiovascular risk prediction score for rheumatoid arthritis; FIB-4, fibrosis-4; GGT, gamma-glutamyl transferase; HDL, high-density cholesterol; hsCRP, high-sensitivity C-reactive protein; Il-6, interleukin-6; LAD, left atrium long-axis diameter; LAV, left atrial volume; LAVI, left atrial volume index; LDL, low-density cholesterol; LSM, liver stiffness measurement; NLR, neutrophil-to-lymphocyte ratio; TG, triglycerides.

**Table 2:** Baseline characteristics of the study participants, including demographic, anthropometric and clinical parameters, medication use, and comorbidities.

Variable	Mean ± SD/Median (IQR)/*n* (%)
Age, years	48.80 ± 8.20
Sex, female	46 (90.2)
Daily alcohol intake, g	0.67 (0.00, 1.33)
Smoking	25 (49)
Disease Duration, years	12 (4.95–20.25)
RF positivity	40 (78.4)
Erosive Disease	26 (50.9)
CDAI	25.00 ± 12.12
HAQ	0.60 (0.28–0.85)
mHAQ > 0.5	20 (39.2)
Medication:	
MTX	46 (90.2)
MTX weekly dose, mg	16.2 (4.4)
MTX cumulative dose, mg	6100 (2400–10,935)
Leflunomide	1 (1.9)
Sulphasalazine	2 (3.9)
Cyclosporine	1 (1.9)
Arechine	1 (1.9)
Biologics	32 (62.7)
Corticosteroids	11 (21.6)
NSAIDS	28 (54.9)
Antihipertensive drugs	11 (21.6)
Statins	3 (5.7)
Body weight, kg	71.24 ± 15.66
Height, cm	164.00 (160.00–168.00)
BMI, kg/m^2^	26.30 ± 5.25
Comorbidites:	
Hyperlipidemia	2 (3.9)
Hypertension	12 (23.5)
Type 2 diabetes	1 (1.9)
Gout	1 (1.9)
Hashimoto disease	3 (5.9)
Chronic kidney disease	1 (1.9)

For continuous variables, values are presented as mean ± SD for normally distributed data or as median (IQR) for non-normally distributed data, and as *n* (%) for categorical variables. Abbreviations: BMI, body mass index; CDAI, clinical disease activity index; HAQ, health assessment questionnaire; mHAQ, modified health assessment questionnaire; MTX, methotrexate; NSAIDs, non-steroidal anti-inflammatory drugs; RF, rheumatoid factor.

## Data Availability

Data are available upon reasonable request.

## Supplementary Materials

The following supporting information can be downloaded at: https://www.mdpi.com/article/10.3390/ijms27156844/s1.

## Abbreviations

The following abbreviations are used in this manuscript:

A	peak A-wave velocity
ACR	American College of Rheumatology
ALT	alanine aminotransferase
APRI	aspartate aminotransferase-to-platelet ratio index
AST	aspartate aminotransferase
BMI	body mass index
CDAI	clinical disease activity index
CI	confidence interval
CV	cardiovascular risk
DMARDs	disease-modifying antirheumatic drugs
E	peak E-wave velocity
EASL	European Association for the Study of the Liver
eGFR	estimated glomerular filtration rate
e’ lat	lateral mitral annular velocity
e’ med	septal mitral annular velocity
ERS-RA	expanded cardiovascular risk prediction score for rheumatoid arthritis
ET-1	endothelin-1
EULAR	European Alliance of Associations for Rheumatology
FIB4	fibrosis-4
GGT	gamma-glutamyl transferase
HDL	high-density lipoprotein
hsCRP	high-sensitivity C-reactive protein
IL-6	interleukin-6
IQR	interquartile range
LAD	left atrium long-axis diameter
LAV	left atrial volume
LAVI	left atrial volume index
LDL	low-density lipoprotein
LF	liver fibrosis
LVDD	left ventricular diastolic dysfunction
LSM	liver stiffness measurement
MASLD	metabolic dysfunction-associated steatotic liver disease
MDRD	modification of diet in renal disease
MTX	methotrexate
mHAQ	modified Health Assessment Questionnaire
mSCORE2	modified systematic coronary risk evaluation 2
NAFLD	non-alcoholic fatty liver disease
NASH	non-alcoholic steatohepatitis
NLR	neutrophil-to-lymphocyte ratio
NSAIDs	non-steroidal anti-inflammatory drugs
OPG	osteoprotegerin
RA	rheumatoid arthritis
RANKL	receptor activator of nuclear factor-κB ligand
ROC	receiver operating characteristic
SBP	systolic blood pressure
SD	standard deviation
SWE	shear wave elastography
TRAIL	TNF-related apoptosis-inducing ligand
T2D	type 2 diabetes
TG	triglycerides
WC	waist circumference
WHO	World Health Organization

## References

[1] Bafna P., Sahoo R.R., Hazarika K., Manoj M., Rungta S., Wakhlu A. (2021). Prevalence of liver fibrosis by Fibroscan in patients on long-term methotrexate therapy for rheumatoid arthritis. Clin. Rheumatol..

[2] Sellami M., Saidane O., Mahmoud I., Tekaya A.B., Tekaya R., Abdelmoula L. (2020). Etiological Features of Liver Involvement in Rheumatoid Arthritis. Curr. Rheumatol. Rev..

[3] Arias-de la Rosa I., Ruiz-Ponce M., Cuesta-López L., Pérez-Sánchez C., Leiva-Cepas F., Gahete M.D., Navarro P., Ortega R., Cordoba J., Pérez-Pampin E. (2023). Clinical features and immune mechanisms directly linked to the altered liver function in patients with rheumatoid arthritis. Eur. J. Intern. Med..

[4] Meng C.C., Chen D.Y., Chen Y.H., Huang W.N., Chen H.H. (2024). Antirheumatic drugs and the risk of nonalcoholic fatty liver disease in patients with rheumatoid arthritis: A nationwide, population-based cohort study. Int. J. Rheum. Dis..

[5] Kim T.Y., Kim J.Y., Sohn J., Lee H.S., Bang S.Y., Kim Y., Kim M.Y., Jeong W.K. (2015). Assessment of Substantial Liver Fibrosis by Real-time Shear Wave Elastography in Methotrexate-Treated Patients With Rheumatoid Arthritis. J. Ultrasound Med..

[6] Feuchtenberger M., Kraus L., Nigg A., Schulze-Koops H., Schäfer A. (2021). Methotrexate does not increase the risk of liver fibrosis in patients with rheumatoid arthritis: Assessment by ultrasound elastography (ARFI-MetRA study). Rheumatol. Int..

[7] Avouac J., Degrave R., Vergneault H., Combier A., Wanono S., Boisson M., Frantz C., Allanore Y. (2022). Risk of liver fibrosis induced by methotrexate and other rheumatoid arthritis medications according to the Fibrosis-4 Index. Clin. Exp. Rheumatol..

[8] Ten Hove M., Smyris A., Booijink R., Wachsmuth L., Hansen U., Alaic L., Faber C., Höltke C., Bansal R. (2024). Engineered SPIONs functionalized with endothelin a receptor antagonist ameliorate liver fibrosis by inhibiting hepatic stellate cell activation. Bioact. Mater..

[9] Owen T., Carpino G., Chen L., Kundu D., Wills P., Ekser B., Onori P., Gaudio E., Alpini G., Francis H. (2023). Endothelin Receptor-A Inhibition Decreases Ductular Reaction, Liver Fibrosis, and Angiogenesis in a Model of Cholangitis. Cell. Mol. Gastroenterol. Hepatol..

[10] Yang B., Lu L., Zhou D., Barbier-Torres L., Steggerda J., Yang H., Yang X. (2022). Regulatory network and interplay of hepatokines, stellakines, myokines and adipokines in nonalcoholic fatty liver diseases and nonalcoholic steatohepatitis. Front. Endocrinol..

[11] Dankbar B., Fennen M., Brunert D., Hayer S., Frank S., Wehmeyer C., Beckmann D., Paruzel P., Bertrand J., Redlich K. (2015). Myostatin is a direct regulator of osteoclast differentiation and its inhibition reduces inflammatory joint destruction in mice. Nat. Med..

[12] Murillo-Saich J.D., Vazquez-Villegas M.L., Ramirez-Villafaña M., Saldaña-Cruz A.M., Aceves-Aceves J.A., Gonzalez-Lopez L., Guma M., Gamez-Nava J.I. (2021). Association of myostatin, a cytokine released by muscle, with inflammation in rheumatoid arthritis: A cross-sectional study. Medicine.

[13] Lin J.Z., Ma J.D., Yang L.J., Zou Y.W., Zhang X.P., Pan J., Li Q.-H., Li H.-G., Yang Z.-H., Wu T. (2022). Myokine myostatin is a novel predictor of one-year radiographic progression in patients with rheumatoid arthritis: A prospective cohort study. Front. Immunol..

[14] Delogu W., Caligiuri A., Provenzano A., Rosso C., Bugianesi E., Coratti A., Macias-Barragan J., Galastri S., Di Maira G., Marra F. (2019). Myostatin regulates the fibrogenic phenotype of hepatic stellate cells via c-jun N-terminal kinase activation. Dig. Liver Dis..

[15] Kaur P., Verma N., Garg P., Ralmilay S., Wadhawan A., Nadda R., Prajapati J., Sharma G., Rathi S., De A. (2024). Myokines are associated with progression, course and mortality in alcohol-associated liver disease. Aliment. Pharmacol. Ther..

[16] Ruiz-Margáin A., Pohlmann A., Lanzerath S., Langheinrich M., Campos-Murguía A., Román-Calleja B.M., Schierwagen R., Klein S., Uschner F.E., Brol M.J. (2023). Myostatin is associated with the presence and development of acute-on-chronic liver failure. JHEP Rep..

[17] Kim J.H., Kang S.H., Lee M., Youn G.S., Kim T.S., Jun B.G., Kim M.Y., Kim Y.D., Cheon G.J., Kim D.J. (2020). Serum Myostatin Predicts the Risk of Hepatocellular Carcinoma in Patients with Alcoholic Cirrhosis: A Multicenter Study. Cancers.

[18] Skladany L., Koller T., Molcan P., Vnencakova J., Zilincan M., Jancekova D., Kukla M. (2019). Prognostic usefulness of serum myostatin in advanced chronic liver disease: Its relation to gender and correlation with inflammatory status. J. Physiol. Pharmacol..

[19] Askin L., Abus S., Tanriverdi O. (2022). Resistin and Cardiovascular Disease: A Review of the Current Literature Regarding Clinical and Pathological Relationships. Curr. Cardiol. Rev..

[20] Corrado A., Colia R., Rotondo C., Sanpaolo E., Cantatore F.P. (2019). Changes in serum adipokines profile and insulin resistance in patients with rheumatoid arthritis treated with anti-TNF-α. Curr. Med. Res. Opin..

[21] Kononoff A., Vuolteenaho K., Hämäläinen M., Kautiainen H., Elfving P., Savolainen E., Cantatore F.P. (2021). Metabolic Syndrome, Disease Activity, and Adipokines in Patients With Newly Diagnosed Inflammatory Joint Diseases. J. Clin. Rheumatol..

[22] Guin A., Sinhamahapatra P., Misra S., Choudhury S., Mazumder S.R., Chatterjee S., Ghosh A. (2019). Incidence and effect of insulin resistance on progression of atherosclerosis in rheumatoid arthritis patients of long disease duration. Biomed. J..

[23] Vuolteenaho K., Tuure L., Nieminen R., Laasonen L., Leirisalo-Repo M., Moilanen E. (2022). NEO-RACo Study Group. Pretreatment resistin levels are associated with erosive disease in early rheumatoid arthritis treated with disease-modifying anti-rheumatic drugs and infliximab. Scand. J. Rheumatol..

[24] Dong Z.X., Su L., Brymora J., Bird C., Xie Q., George J., Wang J. (2013). Resistin mediates the hepatic stellate cell phenotype. World J. Gastroenterol..

[25] Garcia C.C., Piotrkowski B., Baz P., Poncino D., Benavides J., Colombato L., Toso M.L.R., Yantorno S., Descalzi V., Gondolesi G.E. (2022). A Decreased Response to Resistin in Mononuclear Leukocytes Contributes to Oxidative Stress in Nonalcoholic Fatty Liver Disease. Dig. Dis. Sci..

[26] Čustović N., Rašić S. (2022). Relationship of serum adiponectin and resistin levels with the severity of liver fibrosis in patients with chronic hepatitis B. J. Med. Biochem..

[27] Meng Z., Zhang Y., Wei Z., Liu P., Kang J., Zhang Y., Ma D., Ke C., Chen Y., Luo J. (2017). High serum resistin associates with intrahepatic inflammation and necrosis: An index of disease severity for patients with chronic HBV infection. BMC Gastroenterol..

[28] Shen C., Zhao C.Y., Wang W., Wang Y.D., Sun H., Cao W., Yu W.-Y., Zhang L., Ji R., Li M. (2014). The relationship between hepatic resistin overexpression and inflammation in patients with nonalcoholic steatohepatitis. BMC Gastroenterol..

[29] Xu X., Chen J., Yu X., Li Z., Chen J., Fang D., Lin X., Tu H., Xu X., Yang S. (2025). Single-cell analysis identifies RETN^+^ monocyte-derived Resistin as a therapeutic target in hepatitis B virus-related acute-on-chronic liver failure. Gut.

[30] Remuzgo-Martínez S., Genre F., López-Mejías R., Ubilla B., Mijares V., Pina T., Corrales A., Blanco R., Martín J., Llorca J. (2016). Expression of osteoprotegerin and its ligands, RANKL and TRAIL, in rheumatoid arthritis. Sci. Rep..

[31] Ruyssen-Witrand A., Degboé Y., Cantagrel A., Nigon D., Lukas C., Scaramuzzino S., Allanore Y., Vittecoq O., Schaeverbeke T., Morel J. (2016). Association between RANK, RANKL and OPG polymorphisms with ACPA and erosions in rheumatoid arthritis: Results from a meta-analysis involving three French cohorts. RMD Open.

[32] Triguero-Martínez A., Pardines M., Montes N., Ortiz A.M., de la Iglesia-Cedeira A., Valero-Martínez C., Martín J., González-Álvaro I., Castañeda S., Lamana A. (2024). Genetic Variants in RANK and OPG Could Influence Disease Severity and Bone Remodeling in Patients with Early Arthritis. Life.

[33] Vachliotis I.D., Polyzos S.A. (2023). Osteoprotegerin/Receptor Activator of Nuclear Factor-Kappa B Ligand/Receptor Activator of Nuclear Factor-Kappa B Axis in Obesity, Type 2 Diabetes Mellitus, and Nonalcoholic Fatty Liver Disease. Curr. Obes. Rep..

[34] Habibie H., Adhyatmika A., Schaafsma D., Melgert B.N. (2021). The role of osteoprotegerin (OPG) in fibrosis: Its potential as a biomarker and/or biological target for the treatment of fibrotic diseases. Pharmacol. Ther..

[35] Adhyatmika A., Beljaars L., Putri K.S.S., Habibie H., Boorsma C.E., Reker-Smit C., Luangmonkong T., Guney B., Haak A., Mangnus K.A. (2020). Osteoprotegerin is More than a Possible Serum Marker in Liver Fibrosis: A Study into its Function in Human and Murine Liver. Pharmaceutics.

[36] Ciołkiewicz M., Kuryliszyn-Moskal A., Jabłońska E., Ratajczak-Wrona W., Janica J., Samborski W., Klimiuk P.A. (2026). Liver steatosis, selected organokines, and cardiovascular risk markers in rheumatoid arthritis. Front. Endocrinol..

[37] Ferraz-Amaro I., Heras-Recuero E., de Vera-González A., González-Delgado A., Romo-Cordero A., Quevedo-Rodríguez A., Quevedo-Abeledo J.C., Largo R., González-Gay M.Á. (2025). The Fibrosis-4 Index (FIB-4) correlates with cardiovascular risk and insulin resistance in patients with rheumatoid arthritis. Rheumatology.

[38] Saidi A.N., Theel W.B., Burggraaf B., van der Lelij A.J., Grobbee D.E., van Zeben J.D., Beek E.v.d.Z.-V., Rauh S.P., Cabezas M.C. (2025). Metabolic dysfunction-associated steatotic liver disease and cardiovascular risk factors in rheumatoid arthritis. Clin. Rheumatol..

[39] da Silva T.E., Costa-Silva M., Correa C.G., Denardin G., Alencar M.L.A., Coelho M.S.P.H., Muraro-Wildner L., Luiza-Bazzo M., González-Chica D.A., Dantas-Correa E.B. (2018). Clinical Significance of Serum Adiponectin and Resistin Levels in Liver Cirrhosis. Ann. Hepatol..

[40] Kuchay M.S., Martínez-Montoro J.I., Kaur P., Fernández-García J.C., Ramos-Molina B. (2022). Non-alcoholic fatty liver disease-related fibrosis and sarcopenia: An altered liver-muscle crosstalk leading to increased mortality risk. Ageing Res. Rev..

[41] Darabian S., Wade J.P., Kur J., Wade S.D., Sayre E.C., Badii M. (2022). Using FibroScan to Assess for the Development of Liver Fibrosis in Patients With Arthritis on Methotrexate: A Single-center Experience. J. Rheumatol..

[42] Motta F., Di Simone N., Selmi C. (2025). The Impact of Menopause on Autoimmune and Rheumatic Diseases. Clin. Rev. Allergy Immunol..

[43] Zhang Y.Y., Yang N., Zhang H.Y., Ge J.J., Yan S.M., Han D., Qiu Q.Y., Ge W.H., Shu Q. (2025). Steroid hormones and influence of therapeutic drugs in Chinese postmenopausal rheumatoid arthritis patients. Front. Immunol..

[44] Aletaha D., Neogi T., Silman A.J., Funovits J., Felson D.T., Bingham C.O., Birnbaum N.S., Burmester G.R., Bykerk V.P., Cohen M.D. (2010). 2010 Rheumatoid arthritis classification criteria: An American College of Rheumatology/European League Against Rheumatism collaborative initiative. Arthritis Rheum..

[45] European Association for the Study of the Liver (EASL), European Association for the Study of Diabetes (EASD), European Association for the Study of Obesity (EASO) (2024). EASL-EASD-EASO Clinical Practice Guidelines on the management of metabolic dysfunction-associated steatotic liver disease (MASLD). J. Hepatol..

[46] Wai C.T., Greenson J.K., Fontana R.J., Kalbfleisch J.D., Marrero J.A., Conjeevaram H.S., Lok A.S.-F. (2003). A simple noninvasive index can predict both significant fibrosis and cirrhosis in patients with chronic hepatitis C. Hepatology.

[47] MDCALC. https://www.mdcalc.com/calc/3094/ast-platelet-ratio-index-apri.

[48] Sterling R.K., Lissen E., Clumeck N., Sola R., Correa M.C., Montaner J., Sulkowski M.S., Torriani F.J., Dieterich D.T., Thomas D.L. (2006). Development of a simple noninvasive index to predict significant fibrosis in patients with HIV/HCV coinfection. Hepatology.

[49] MDCALC. https://www.mdcalc.com/calc/2200/fibrosis-4-fib-4-index-liver-fibrosis.

[50] Verity Research. https://www.verityresearch.org/cvd-risk-calculator.

[51] Ferraioli G., Wong V.W., Castera L., Berzigotti A., Sporea I., Dietrich C.F., Choi B.I., Wilson S.R., Kudo M., Barr R.G. (2018). Liver Ultrasound Elastography: An Update to the World Federation for Ultrasound in Medicine and Biology Guidelines and Recommendations. Ultrasound Med. Biol..

[52] Bauer D.J., Matic V., Mare R., Maiocchi L., Chromy D., Müllner-Bucsics T., Mandorfer M., Mustapic S., Sporea I., Ferraioli G. (2023). Point Shear Wave Elastography by ElastPQ for Fibrosis Screening in Patients with NAFLD: A Prospective, Multicenter Comparison to Vibration-Controlled Elastography. Ultraschall Med..

[53] Lang R.M., Badano L.P., Mor-Avi V., Afilalo J., Armstrong A., Ernande L., Flachskampf F.A., Foster E., Goldstein S.A., Kuznetsova T. (2015). Recommendations for Cardiac Chamber Quantification by Echocardiography in Adults: An Update from the American Society of Echocardiography and the European Association of Cardiovascular Imaging. J. Am. Soc. Echocardiogr..

[54] Nagueh S.F., Smiseth O.A., Appleton C.P., Byrd B.F., Dokainish H., Edvardsen T., Flachskampf F.A., Gillebert T.C., Klein A.L., Lancellotti P. (2016). Recommendations for the Evaluation of Left Ventricular Diastolic Function by Echocardiography: An Update from the American Society of Echocardiography and the European Association of Cardiovascular Imaging. J. Am. Soc. Echocardiogr..

