# Supplementary material for: The Liver–Heart Axis in Rheumatoid Arthritis: Associations of Liver Fibrosis, Organokines, Endothelin-1, and Cardiovascular Risk
Source: Int J Mol Sci. 2026 Jul 30;27(15):6844. doi: 10.3390/ijms27156844 (PMC13466673; doi:10.3390/ijms27156844)
Supplement: Supplementary file 1 [file ijms-27-06844-s001.zip › ijms-4462956-supplementary.pdf]

SUPPLEMENTARY MATERIAL

Table S1. Results of univariable linear regression analyses for APRI and FIB-4

	**APRI**			**FIB-4**		
	**β (95% CI)**	**Std β**	**p**	**β (95% CI)**	**Std β**	**p**
Sex, female	0.01 (-0.12.1 to 0.14)	0.02	0.87	-0.05 (-0.42 to 0.33)	-0.04	0.81
Age, years	0.001 (-0.003 to 0.006)	0.08	0.57	0.03 (0.01 to 0.04)	0 .54	<0.001
MTX use	0.03 (-0.10 to 0.16)	0.07	0.62	-0.07 (-0.44 to 0.31)	-0.05	0.72
Mean MTX weekly dose	0.004 (-0.006 to 0.01)	0.12	0.42	0.02 (-0.007 to 0.05)	0.21	0.15
Cumulative MTX dose	0.000003 (-0.000004 to 0.000009)	0.12	0.39	0.00001 (-0.000009 to 0.00003)	0.15	0.30
Biologics	-0.03 (-0.11 to 0.06)	-0.10	0.47	-0.13 (-0.36 to 0.09)	-0.17	0.24
Steroids	-0.04 (-0.13 to 0.06)	-0.12	0.42	-0.31 (-0.57 to -0.06)	-0.33	0.02
NSAIDs	-0.03 (-0.11 to 0.05)	-0.01	0.47	0.05 (-0.17 to 0.27)	0.06	0.66
Alcohol g/24h	-0.005 (-0.02 to 0.006)	-0.13	0.38	-0.007 (-0.04 to 0.02)	-0.07	0.65
hsCRP, mg/dL	0.004 (0.002 to 0.007)	0.48	<0.001	-0.005 (-0.01 to 0.002)	-0.20	0.16
IL-6, pg/mL	0.001 (-0.0002 to 0.003)	0.24	0.09	0.0008 (-0.004 to 0.005)	0.05	0.71
NLR	-0.01 (-0.06 to 0.03)	-0.08	0.57	0 .03 (- 0.10 to 0.15)	0 .07	0.65
Endothelin-1, pg/mL	-0.05 (-0.14 to 0.04)	-0.15	0.28	-0.11 (-0.38 to 0.15)	-0 .12	0.39
Myostatin, ng/mL	0.01 (-0.02 to 0.04)	0.09	0.53	0.05 (-0.04 to 0.1)	0.17	0.24
Resistin, ng/mL	-0.002 (-0.01 to 0.007)	-0.06	0.69	-0.01 (-0.04 to 0.02)	-0 .12	0.40
Osteoprotegerin, pg/mL	0.0003 (-0.000 2 to 0.0007)	0.19	0.19	0.001(-0.0002 to 0.002)	0.24	0.09

Abbreviations:, hsCRP, high sensitivity C-reactive protein, IL-6, interleukin-6, MTX, methotrexate, NLR, Neutrophil-to-Lymphocyte Ratio, NSAIDS, non-steroidal anti-inflammatory drugs

Table S2. Results of univariable linear regression analyses for LSM

	**LSM**		
	**β (95% CI)**	**Std β**	**p**
Sex, female	-0.50 (-1.56 to 0.55)	-0.14	0.34
Age, years	0.02 (-0.02 to 0.06)	0.13	0.37
MTX use	-0.77 (-1.81 to 0.27)	-0.21	0.14
Mean MTX weekly dose	-0.002 (-0.08 to 0.07)	-0.01	0.95
Cumulative MTX dose	-0.00001 (0.00006 to 0.00004)	-0.06	0.70
Biologics	-0.35 (-1.00 to -0.30)	-0.15	0.28
Steroids	0.29 (-0.47 to 1.06)	0.11	0.45
NSAIDs	-0.10 (-0.74 to 0.54)	-0.05	0.76
Alcohol g/24h	-0.07 (-0.15 to 0.02)	-0.20	0.15
hsCRP, mg/dL	-0.01 (-0.00 to 0.01)	-0.15	0.31
Il-6, pg/mL	0.006 (-0.007 to 0.02)	0.13	0.37
NLR	0.0007 (-0.36 to 0.36)	0.0006	0.99
ET-1, pg/mL	0.12 (-0.64 to 0.87)	0.04	0.76
Myostatin, ng/mL	0.26 (0.02 to 0.51)	0.29	0.04
Resistin, ng/mL	-0.14 (-0.23 to -0.06)	-0.44	0.002
Osteoprotegerin, pg/mL	0.001 (-0.003 to 0.005)	0.08	0.57

Abbreviations:, hsCRP, high sensitivity C-reactive protein, IL-6, interleukin-6, MTX, methotrexate, NLR, Neutrophil-to-Lymphocyte Ratio, NSAIDS, non-steroidal anti-inflammatory drugs

Table S3. Results of the logistic regression analysis for LVDD predictors.

**Predictor**	**OR**	**95% CI**	**p**
Age, years	1.10	0.99 to 1.22	0.06
Sex, female	0.32	0.05 to 2.21	0.25
Daily alcohol dose	0.99	0.80 to 1.22	0.91
Methotrexate	0.97	0.10 to 9.80	0.98
Mean MTX weekly dose	1.03	0.87 to 1.21	0.72
MTX cumulative dose	1.00	1.00 to 1.00	0.11
Biologics	0.87	0.21 to 3.57	0.84
Steroids	0.89	0.16 to 4.95	0.89
NSAIDS	3.81	0.72 to 20.16	0.12
hsCRP	0.92	0.77 to 1.10	0.37
NLR	1.80	0.84 to 3.85	0.13
IL-6	0.99	0.95 to 1.03	0.54
Endothelin-1, pg/mL	2.51	0.52 to 12.03	0.25
Myostatin, ng/mL	1.35	0.80 to 2.27	0.26
Resistin, ng/mL	0.77	0.58 to 1.02	0.07
Osteoprotegerin, pg/mL	1.01	0.99 to 1.02	0.08
APRI	2.00	0.02 to 269.26	0.78
FIB-4	2.41	0.46 to 12.66	0.30
LSM	1.91	0.83 to 4.41	0.13

Abbreviations: APRI, aspartate aminotransferase-to-platelet ratio index, FIB-4, fibrosis-4, hsCRP, high sensitivity C-reactive protein, IL-6, interleukin-6, LSM, liver stiffness measurement, MTX, methotrexate, NLR, Neutrophil-to-Lymphocyte Ratio, NSAIDS, non-steroidal anti-inflammatory drugs
